# A Dermatological Mystery Solved

**Published:** 1902-11-29

**Authors:** 


### A DERMATOLOGICAL MYSTERY SOLVED.

Some very curious cases of blue discolouration of the skin of the feet, occurring in patches and especially between the toes, have lately been reported. A very good example was shown at one of the afternoon consultations at the London Polyclinic some time ago, and three cases are related by Dr. Arthur Hall, of Sheffield, in the November number of the *British Journal of Dermatology*. The discolouration seems to have been similar in all the cases, the colour produced being of that blue-green tint which goes by the name of peacock-blue. It could not be removed by washing, and, in the Polyclinic case, even firm friction with spirits of wine failed to remove the stain, while no tinge was given to the spirit. The colour was absolutely unlike any ordinary pathological product, and in view of its very patchy distribution, it was not unnatural to imagine that the colour had been applied, and that the case was but another example of those strange freaks sometimes played by patients suffering from hysteria. In Dr. Hall's cases one of the patients was a girl aged 16, another a married woman aged 50, and the third a woman aged 30. In all of these the skin between the toes was the part principally affected. Meanwhile a case had come under observation in which a similar discolouration was present in the axillæ. This patient was wearing a dark purple blouse, and as the axilla alone were affected, it seemed probable that the staining had resulted from some reaction between the perspiration and the dye. On further investigation it was found that the stockings worn by the patient who had come last under observation were cheap black ones, and on soaking portions of them respectively in a weak acid solution, a weak alkaline solution, and distilled water, in each case maintained at the body heat, the acid solution soon became strongly coloured peacock-blue. Hence it may be presumed that the discolouration which had puzzled so many observers was the result of the reaction of the acid perspiration of the parts affected and the dye of the stockings.

